# The road to the structure of the mitochondrial respiratory chain supercomplex

**DOI:** 10.1042/BST20190930

**Published:** 2020-04-20

**Authors:** Nikeisha J. Caruana, David A. Stroud

**Affiliations:** Department of Biochemistry and Molecular Biology, Bio21 Molecular Science and Biotechnology Institute, University of Melbourne, Parkville 3052 Victoria, Australia

**Keywords:** mitochondria, oxidative phosphorylation, structural biology

## Abstract

The four complexes of the mitochondrial respiratory chain are critical for ATP production in most eukaryotic cells. Structural characterisation of these complexes has been critical for understanding the mechanisms underpinning their function. The three proton-pumping complexes, Complexes I, III and IV associate to form stable supercomplexes or respirasomes, the most abundant form containing 80 subunits in mammals. Multiple functions have been proposed for the supercomplexes, including enhancing the diffusion of electron carriers, providing stability for the complexes and protection against reactive oxygen species. Although high-resolution structures for Complexes III and IV were determined by X-ray crystallography in the 1990s, the size of Complex I and the supercomplexes necessitated advances in sample preparation and the development of cryo-electron microscopy techniques. We now enjoy structures for these beautiful complexes isolated from multiple organisms and in multiple states and together they provide important insights into respiratory chain function and the role of the supercomplex. While we as non-structural biologists use these structures for interpreting our own functional data, we need to remind ourselves that they stand on the shoulders of a large body of previous structural studies, many of which are still appropriate for use in understanding our results. In this mini-review, we discuss the history of respiratory chain structural biology studies leading to the structures of the mammalian supercomplexes and beyond.

## Introduction

Mitochondria produce the vast majority of the ATP used by eukaryotic life and as such have been frequently labelled the ‘powerhouse of the cell’ by popular culture and academics alike [1]. Production of ATP occurs on the F_o_F_1_-ATP synthase, a molecular motor that draws its power from a proton gradient created across the inner mitochondrial membrane by the electron transport chain (also known as the respiratory chain) [2]. The coupling of ATP production to respiration in this way is known as oxidative phosphorylation (OXPHOS). The electron transport chain consists of four multi-protein membrane complexes; Complex I (CI, NADH:ubiquinone oxidoreductase), Complex II (CII, succinate:ubiquinone oxidoreductase), Complex III (CIII, cytochrome *bc1* complex) and Complex IV (CIV, cytochrome *c* oxidase). Acetyl coenzyme A derived from the metabolism of sugars, fats and amino acids is oxidised by enzymes in the tricarboxylic acid (TCA) cycle, and electrons transferred to carriers such as nicotinamide adenine dinucleotide (NADH) and succinate. In turn, NADH and succinate are oxidised by Complexes I and II to reduce ubiquinone (Coenzyme Q; CoQ), which is, in turn, oxidised by Complex III to reduce cytochrome *c.* The electron transport chain concludes with cytochrome *c* being oxidised by Complex IV to reduce O_2_ to water [3]. Electron transport through Complexes I, III and IV drives the pumping of protons out of the mitochondrial matrix and generates an electrochemical gradient, which is used by the F_o_F_1_-ATP synthase to power ATP synthesis. Although Complex II does not contribute to the generation of the proton gradient directly, it oxidises succinate to fumarate thereby reducing ubiquinone to ubiquinol and therefore increasing the electrons available to Complexes III and IV [4]. Mitochondria contain their own DNA, known as mitochondrial DNA (mtDNA), which in mammals encodes 13 proteins, all of which are membrane-spanning subunits found in the OXPHOS complexes — 7 in CI, one in CIII, three in CIV and two in the F_o_F_1_-ATP synthase. During the biogenesis of the individual complexes, these coalesce with more than 70 other subunits encoded by nuclear DNA (nDNA) to form the mature complexes [5]. Highlighting the importance of this system, mutations in all 13 mtDNA encoded genes and many of the nuclear genes encoding subunits and critical assembly factors cause mitochondrial disease, a group of inherited disorders of the OXPHOS system with a birth prevalence of 1 : 5000 [6,7].

The structural integrity of the individual complexes as well as their interaction is of vital importance for efficient OXPHOS. This is elegantly highlighted in the many studies of mitochondrial disease patients who harbour mutations in the genes encoding OXPHOS subunits (catalogued in [6]). Of recent interest is the stable interaction of Complexes I, III and IV which was originally observed during the development of native electrophoresis techniques [8]. Although the association of these complexes into stable assemblies known as respiratory chain supercomplexes (or respirasomes) was initially controversial, the phenomenon has since been observed in multiple organisms using a multitude of approaches. The function of these enormous membrane protein complexes (1.7 MDa consisting of 80 different subunits [9,10]), remains a subject of ongoing debate (for excellent reviews on this topic see [11,12–14]). The major roles proposed for the supercomplexes include the stabilisation of individual complexes [15] and the channelling of substrates [16], both of which would provide a level of protection against the production of reactive oxygen species (ROS), by-products of inefficient OXPHOS.

**Figure 1.**
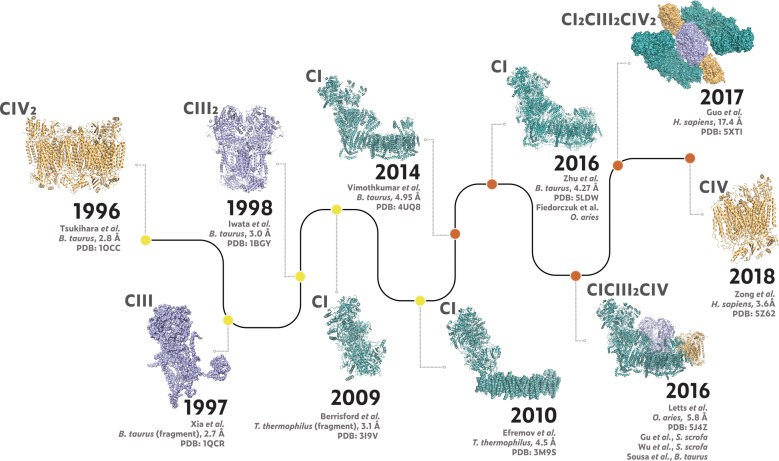
Timeline for the identification of key structures for each of the three respiratory chain complexes present in the supercomplex. Structures solved using X-ray crystallography are coloured by yellow nodes, with Cryo-EM structures coloured by orange nodes. Structures which had low-quality density maps (PDB:1QCR and 5XTI) or only existing as an alpha-helical model (membrane region of PDB:3M9S) are depicted using the surface representation, whereas high-resolution structures are shown using the ribbon representation. Note: the CI_2_/CIII_2_/CIV_2_ megacomplex structure is a top view, whereas other structures are shown as side views.

High-resolution structures of the OXPHOS complexes have been critical to our understanding of their function in respiration, however, these structures also proved a valuable resource for researchers interested in the mechanisms of OXPHOS complex assembly and how defective OXPHOS might lead to disease. Our laboratory has benefitted immensely from the work of structural biologists, as we have found the mapping of mass-spectrometry derived data onto the 3D structures of OXPHOS complexes helpful for understanding the roles of specific subunits and assembly factors [17–23]. Complete high-resolution structures now exist for four of the five OXPHOS complexes, as well as multiple variations of the respiratory chain supercomplex. Although X-ray crystallography structures for the intact Complexes III and IV were published in the 1990s [24–28], the complete structures of Complex I and the respiratory chain supercomplex necessitated the development of Cryo-EM technology. Many structures solved by Cryo-EM utilise existing high-resolution structural data of individual subunits, fragments or subcomplexes to build starting models [29,30] and Complex I and the respiratory chain supercomplexes have been no exception to this rule. As a result, the impressive Cryo-EM structures of recent years stand on the shoulders of many other structural studies. This mini-review highlights the structural discoveries made on the road to the recent structures of the mammalian respiratory chain supercomplex (Figure 1). We also hope that this mini-review can act as a guide for other non-structural biologists in choosing the appropriate structure to use in interpreting their data.

## Complex IV: cytochrome *c* oxidase

Complex IV consists of 14 subunits [25,31] including three subunits encoded by mtDNA, all of which have direct homologues in yeast and bacteria. Mammalian Complex IV contains 11 subunits encoded by nDNA, some of which are present as multiple isoforms with varying tissue expression [5,32–34]. Complex IV is the only OXPHOS complex known to harbour tissue-specific subunits, which are suspected to tailor the enzyme activity of the complex to the needs of a specific tissue [32]. Complex IV was the first OXPHOS complex to yield a low-resolution structure, and was an early beneficiary of electron microscopy, with the technique paired with 2D membrane crystals to reveal a monomeric bovine Complex IV, highlighting its basic topological features [35]. The first high-resolution structure was of the bovine Complex IV, the first for any of the mammalian OXPHOS complexes, published in 1996 at 2.8 Å (PDB: 1OCC; Figure 1) using X-ray crystallography [25]. In their structure, Yoshikawa and co-workers [25] described a dimer with each monomer containing 13 different subunits. Although many structures of mammalian Complex IV have been solved since [36,37], the overall state of the structure remained relatively unchanged until 2018. In 2018, Yang and co-workers [38] combined structural and biochemical approaches to propose that the dimerisation of Complex IV observed in most previous structural studies occurred during purification of the complex. Their 3.3 Å structure of human Complex IV (PDB: 5Z62; Figure 1) was isolated from the commonly used human embryonic kidney (HEK293) cell line and solved by Cryo-EM. The human Complex IV structure was modelled from a recent 1.5 Å bovine Complex IV structure solved by X-ray crystallography [39], revealing a monomeric complex consisting of 14 unique subunits. Importantly, this is the first structure of Complex IV to contain NDUFA4, initially assigned as a Complex I subunit [40] but reassigned to Complex IV in 2012 based on biochemical studies [31]. In their structure, Yang and co-workers found NDUFA4 to be located at the site of Complex IV dimerisation and suggest it and a cardiolipin molecule also present in their structure is displaced by the cholic acid salt used in crystallisation buffers. This conclusion is supported by the work of Shinzawa-Itohet al. [41], who recently solved the X-ray structure of both oxidised and reduced forms of bovine Complex IV monomers (at 1.85 Å and 1.95 Å respectively; PDB: 6JY3 and 6JY4) using novel synthetic detergents. Although NDUFA4 is absent from their structures, they found that the activity of Complex IV is lower for the dimer than for the monomer, suggesting the latter to be the active form of the complex [41]. Other arguments against dimeric Complex IV being functionally relevant include the presence of monomeric Complex IV in all structures of the supercomplex (discussed below), the lack of dimeric Complex IV in prokaryotes [42], and early detergent studies demonstrating the influence of different detergents on Complex IV dimerisation [43]. Moreover, it has recently been proposed that there is a continuous transition between the monomer and dimer of Complex IV via reversible phosphorylation, with the dimeric form induced by high ATP/ADP ratios, thereby inhibiting respiration and preventing ROS [44]. Since many conclusions as to the molecular mechanism of Complex IV activity have been drawn from X-ray structures of the dimeric complex [25,45,46], the physiological relevance of the dimer is an important aspect to explore in future work.

## Complex III: cytochrome *bc_1_* complex

Complex III is the central component of the respiratory chain, coupling the transfer of electrons from coenzyme Q (passed from Complexes I and II) to cytochrome *c* in Complex IV and contributing to the generation of the proton gradient across the mitochondrial membrane. Of the three proton-pumping complexes, Complex III has the least number of subunits, one encoded by mtDNA and 10 encoded on nDNA [5,47]. An interesting quirk of Complex III is the post-translational cleavage of the protein product of the *UQCRFS1* gene into mature UQCRFS1 (also known as the Rieske iron–sulfur protein) and an N-terminal fragment known as UQCRFS1N (or subunit 9), both of which are present in the mature complex. In mammals, cleavage is thought to occur after insertion of the full-length protein into the complex through matrix processing peptidase activity present in the Complex III subunits UQCRC1 and UQCRC2 [47]. Like for Complex IV, 2D crystals and electron microscopy combined with biochemical analysis gave early insights into the structure and topology of Complex III [26–28], however, atomic resolution structures did not emerge until the 1990s. A partial structure of the complex isolated from bovine heart mitochondria was determined in 1997 by Deisenhofer and co-workers [27] using X-ray crystallography (PDB: 1QCR; Figure 1), revealing 5 of the 11 subunits in full, and small regions of two other subunits. The structure described a symmetric dimer, consistent with previous biochemical studies [28,48,49]. The complete bovine Complex III was revealed in 1998, with Iwata et al. [28] describing a dimer of 11-subunit monomers at 3.0 Å (PDB: 1BGY; Figure 1). Like the other complexes, there have been a multitude of subsequent structures isolated from various organisms under different conditions designed to investigate the molecular mechanism underpinning the enzymes function [49,50]. Most recently, researchers have focused on the central role of Complex III in the supercomplex (discussed below) although it is worthwhile here to note the recent proposal of Yang and co-workers, who suggest based on re-analysis of high-resolution X-ray structures [51,52] that the Complex III dimer contains only a single UQCRFS1N fragment [53]. This would make it an asymmetric dimer of 11 and 10 individual subunits, with implications for Complex III function [53] that need to be clarified in future structural studies. Moreover, this finding is in line with recent data concerning the role of Complex III assembly factor TTC19 in proteolytic removal of the UQCRFS1N fragment, which the authors found was necessary to maintain enzyme function [54].

## NADH:ubiquinone oxidoreductase — Complex I

Complex I (mitochondrial NADH:ubiquinone oxidoreductase) is one of the largest and most complicated enzymes within mammalian cells. It is responsible for oxidising NADH in the mitochondrial matrix, regenerating NAD^+^ to sustain the TCA cycle and fatty acid oxidation, as well as contributing to the proton gradient across the inner membrane. Mammalian Complex I consists of 45 subunits including 7 encoded by mtDNA and 37 by nDNA, one of which is present twice in the complex [31,55,56]. While the high-resolution structures of the complete or near-complete Complexes III and IV were determined by X-ray crystallography in the 1990s, the sheer scale of Complex I necessitated a combination of X-ray crystallography, Cryo-EM and improved sample preparation techniques to reveal its high-resolution structure. More than any other complex, structural determination of mammalian Complex I relied on knowledge gleaned from the ‘minimal' fungal and bacterial structures [57]. Of the 45 subunits in the mammalian complex, homologues for all 7 mtDNA encoded subunits and 7 nDNA encoded ‘core' subunits are found in bacterial Complex I [57,58]. All active centres are located in these 14 subunits, thus the remaining 30 subunits, most of which are unique to multicellular eukaryotic life, are known as supernumerary or accessory subunits [17,59,60]. Like for the other proton-pumping complexes, 2D crystals (isolated from the fungus *Neurospora crassa*) and electron microscopy contributed to our understanding of Complex I topology, specifically its orientation in the membrane [61,62] and its characteristic ‘L’ shape — a membrane-embedded domain with a matrix exposed hydrophilic domain. With the development and rise of Cryo-EM, low-resolution 3D structures of bovine, *N. crassa, Yarrowia lipolytica* and bacterial complexes were produced [63–66]. There were many notable X-ray crystallography studies that were instrumental in aiding our high-resolution understanding of Complex I, many of these benefitting from methods developed in the previous decades for biochemical purification of specific subdomains. Berrisford and Sazanov [29] were first to solve the structure of the hydrophilic domain of the bacterial (*Thermus thermophilus*) complex (PDB: 3I9 V; Figure 1), and used this as a pathway to the 4.5 Å structure of the entire complex in 2010 (PDB: 3M9S; Figure 1) [30]. This was shortly followed by Brandt and co-workers [67], who were first to solve the structure of a complete eukaryotic Complex I, presenting the fungal *Y. lipolytica* structure at 6.3 Å (subsequently improved to 3.6 Å in 2015 [68]). While these structures undoubtedly yielded important insights into the molecular mechanisms underpinning Complex I function, a high-resolution structure of the mammalian complex necessitated the development of more advanced techniques, in particular improvements in sample preparation and the advent of direct electron detectors for Cryo-EM [69]. On the back of these developments, in 2014 Hirst and co-workers [55] unveiled a 4.95 Å resolution structure of the bovine Complex I (PDB: 4UQ8; Figure 1), the first respiratory chain complex solved using Cryo-EM techniques using current instrumentation and methodology. This structure was responsible for many notable advances in our understanding of Complex I structure and function, in particular, the locations of 18 of the 30 accessory subunits found in mammals, and the presence of two copies of the NDUFAB1 subunit. Improvements in purification strategies and continual development of Cryo-EM techniques saw in 2016 the Hirst and Sazanov groups producing structures for entire bovine [70] (PDB: class 1: 5LDW at 4.27 Å; Figure 1) and ovine [71] (PDB: 5LNK at 3.9 Å) complexes, revealing all 45 subunits at atomic resolution. Computational sorting of particles gathered from the bovine data revealed three different structural classes, giving the first insights into how the conformation of the enzyme changes as it switches from active to inactive states [70]. With the structure of the entire complex now resolved, the focus has turned to determining structures of specific states for understanding the mechanisms underpinning Complex I function, as well as the roles of specific subunits and assembly factors. In the case of the former, structures for both an active and inactive form of the *Mus musculus* Complex I were solved at 3.3 Å resolution [72] and the inactive form of the bovine enzyme at 4.1 Å [73] revealing conformational variations in the membrane domain and allowing for the development of further models describing the proton-pumping mechanism, and improving our understanding of how Complex I function recovers following deactivation during hypoxia. For the latter, the functions of the accessory subunits are not yet clear. While the lack of homologues for these proteins in bacteria suggest they possess no catalytic roles, most seem to be critical for assembly and or stability of the complex [17]. This was elegantly shown by Zickermann and co-workers [74], who in 2019 solved the structures of Complex I isolated from yeast (*Y. lipolytica*) mutants lacking accessory subunits NDUFS4 and NDUFS6. Mutations in the genes encoding these subunits are known to lead to the turnover of these proteins leading to mitochondrial disease [6,17], thus the structures give important pathological insights. Notably, this study is also the first to present a structure of an assembly intermediate for Complex I, with the authors revealing a homologue of the assembly factor NDUFAF2 bound to an incomplete Complex I isolated from cells lacking NDUFS6. The authors conclude that assembly factor NDUFAF2 binds to the position eventually occupied by the subunit NDUFA12, preventing the binding of NDUFS6, preventing reverse electron flow and production of ROS from a partially assembled complex. We eagerly await further structures of assembly intermediates for Complex I and the other OXPHOS complexes over the coming years.

## The respiratory chain supercomplexes

The development of native gel electrophoresis (primarily blue native polyacrylamide gel electrophoresis; BN-PAGE) in the 1990s [8] led to the identification of many enormous (1.5–2 MDa) structures containing various configurations of Complexes I, III and IV known as supercomplexes. The supercomplex containing a single unit of Complex I, the Complex III dimer and a single unit of Complex IV (CI/III_2_/IV) is typically the most abundant assembly observed using native electrophoresis techniques, and therefore is considered the base functional unit of the supercomplex or ‘respirasome'. The critical roles speculated for the respirasome include improved channelling of CoQ and cytochrome *c* between the complexes, increased complex stability and protection from ROS [12,13]. Though the bioenergetic advantage conferred by substrate channelling [75–77], is arguably the most often cited of proposed functions, the concept is disputed based on both structural and biochemical evidence [9,10,15,78–81]. Indeed, the supercomplexes themselves were initially criticised for being artefacts of analysis in the presence of ionic detergents, however, evidence for the existence of the supercomplexes increased with their identification without detergents [15], the advances in Cryo-EM techniques, which in the 2010s led to the emergence of multiple supercomplex structures, and recent observations of the supercomplexes *in situ* using cryo-electron tomography (Cryo-ET) [82].

The first supercomplex structure revealed in atomic detail was the ovine CI/III_2_/IV respirasome in 2016. Sazanov and co-workers [9] presented structures for two forms of the respirasome — ‘loose' and ‘tight', as well as the CI/III_2_ supercomplex at high resolution (PDBs: 5J4Z, 5J7Y and 5J8K respectively; Figure 1). These were closely followed by structures of the porcine respirasome [10,80] (PDBs: 5GPN and 5GUP) from the Yang group and bovine [77] (PDB: 5LUF) respirasome from Kühlbrandt and co-workers. While the porcine structures have higher resolutions (4 Å for 5GUP vs 5.8 Å and 9 Å for the ovine and bovine structures) it is important to note that they contain problems in the assignment of subunits and cofactors (discussed in [11,13]). While none of the structures revealed new subunits specific to the supercomplex, they led to many interesting observations. All respirasomes show the membrane arm of Complex I curving around the Complex III dimer, with a monomer of Complex IV located between Complexes I and III at the ‘toes' of Complex I. The positions of Complex IV varies substantially in the porcine and ‘tight' and ‘loose' ovine structures, likewise Complex III is found to be rotated in the bovine and second porcine structure, which may indicate the capture of different functional states. Indeed, the ability to differentiate different ‘classes' within a single sample preparation is a major feature of Cryo-EM — for example, it is evident in the first ovine structure that Complex IV subunit COX7A switches between Complex IV and Complex I in the ‘tight' and loose' forms of the respirasome [9]. Using an improved isolation strategy, Sazanov and co-workers [83] continued this approach, identifying four distinct forms of the ovine CI/III_2_ supercomplex structure at high resolution (3.8 Å) (see Letts et al. [83] for relevant PDB accession numbers). These structures describe striking differences in the conformations of subunits describing how one complex might be able to affect change on another within the same respirasome. Moreover, the authors show that CoQ is not equally able to access its sites in Complex III, suggesting its oxidation is a major rate-limiting step. While these structures have led to other important mechanistic and structural insights, the structures also provide strong evidence against the concept of substrate channelling, since they show the CoQ binding sites of Complexes I and III to be separated by ∼100 Å, and reveal no steric hinderance for the diffusion of cytochrome *c* between Complexes III and IV [9,10,80,83].

Finally, in 2017 Yang and co-workers [79] were able to isolate a higher-order assembly of the respirasome (CI_2_III_2_IV_2_) which they term megacomplex. To resolve this structure, the authors first solved the structure of the major CI/III_2_/IV respirasome purified from human embryonic kidney cells (HEK293), making this both the first structure of a human supercomplex and the highest resolution (3.9 Å; PDB: 5XTH) and most complete for any mammalian supercomplex. The megacomplex itself is of significantly lower resolution (17.4 Å; PDB: 5XTI; Figure 1) and built from the human CI/III_2_/IV respirasome structure, however, it reveals a circular architecture with the Complex III dimer in the centre and each monomer contacting a single unit of Complexes I and IV. While their human origin will for many researchers make them useful structural models, like for the porcine respirasome structures these contain some problems with the assignment of features (discussed in [11]). Moreover, the authors also suggest that two units of Complex II could be modelled into the megacomplex structure between the distal ends of Complexes I and IV. Although they provided no evidence for this in terms of unassigned density, they justified this speculative model based on previous biochemical studies [75,84]. While a complex containing all the complexes of the electron transport chain is a tantalising idea, further experimental work will be needed to clarify if this is reflective of the reality inside the cell.

## Perspectives

- Structural characterisation of the mitochondrial respiratory chain complexes has been important for understanding their function. High-resolution structures for Complexes III and IV were determined by X-ray crystallography, however, the size of Complex I and the supercomplexes necessitated advances underpinning modern Cryo-EM. As a result of this, a flurry of structures emerged in the 2010s culminating with five separate publications in the space of a year describing the mammalian supercomplex in high-resolution and showing the complexes in multiple states. This led to important insights into respiratory chain function.
- Cryo-EM typically uses data gathered under more physiological conditions than for X-ray crystallography and reveals multiple structural forms, leading to a deeper understanding of conformational changes. For the supercomplex, this has led to hypotheses concerning cross-talk between its components.
- Combining this with mammalian gene-editing techniques and the recent ability to purify the respiratory chain complexes from human cell lines opens the door for a new era of structure–function studies, particularly the elucidation of assembly intermediates and disease states. Future development of single-particle analysis and Cryo-ET techniques will further open the door to a new era of structure-function studies.

## Open Access

Open access for this article was enabled by the participation of the University of Melbourne in an all-inclusive *Read & Publish* pilot with Portland Press and the Biochemical Society under a transformative agreement with CAUL.

## Abbreviations

mtDNA: mitochondrial DNA
NADH: nicotinamide adenine dinucleotide
nDNA: nuclear DNA
OXPHOS: oxidative phosphorylation
ROS: reactive oxygen species
TCA: tricarboxylic acid
